# Factors Related to Mortality in Patients with COVID-19 during the Early Phase of the Pandemic in Japan: An Observational Study Using the Osaka Prefectural Novel Coronavirus Response Status Management System

**DOI:** 10.31662/jmaj.2023-0179

**Published:** 2024-06-17

**Authors:** Kyoko Kondo, Asae Suita, Satoko Ohfuji, Emiko Mukai, Tetsuo Kase, Wakaba Fukushima

**Affiliations:** 1 Management Bureau, Osaka Metropolitan University Hospital, Osaka, Japan; 2 Research Support Platform, Osaka Metropolitan University Graduate School of Medicine, Osaka, Japan; 3 Department of Public Health, Osaka Metropolitan University Graduate School of Medicine, Osaka, Japan; 4 Research Center for Infectious Disease Sciences, Osaka Metropolitan University Graduate School of Medicine, Osaka, Japan; 5 Osaka International Research Center for Infectious Diseases, Osaka Metropolitan University, Osaka, Japan

**Keywords:** COVID-19, epidemiology, Japan, underlying disease, mortality

## Abstract

**Introduction:**

Elucidating the epidemiological picture in the early phase of a pandemic is crucial to strengthening preparedness and public health responses to future emerging infectious diseases. Using data from the “Osaka Prefectural Novel Coronavirus Response Status Management System,” we evaluated factors associated with mortality among patients with novel coronavirus disease 2019 (COVID-19) in Osaka Prefecture, Japan.

**Methods:**

The study periods were from January 29 to June 13, 2020 (first surge), from June 14 to October 9, 2020 (second surge), and from October 10 to December 24, 2020 (up to the middle of the third surge). The odds ratios (ORs) and 95% confidence intervals (95% CIs) for mortality were calculated using logistic regression models.

**Results:**

Of the 14,864 patients with COVID-19 (8,207 men, 6,657 women) registered, 297 (2%) died. The ORs for mortality were significantly higher in men (OR = 2.00, 95% CI = 1.54-2.60) than in women, in 70- to 79-year-olds (OR = 25.4, 95% CI = 16.8-38.2) and ≥80-year-olds (OR = 78.1, 95% CI = 53.3-114) than in 0- to 69-year-olds (*P* for trend < 0.001), and in those with underlying diseases (OR = 1.74, 95% CI = 1.34-2.27) than in those without. The ORs for the second surge (OR = 0.42, 95% CI = 0.31-0.57) and third surge (OR = 0.41, 95% CI = 0.29-0.58) decreased compared with the first surge. Detailed evaluation of underlying diseases by time period showed that “Diseases of the blood and blood-forming organs and certain disorders involving immune mechanisms,” “Endocrine, nutritional, and metabolic diseases,” “Diseases of the genitourinary system,” and “Diseases of the respiratory system” were associated with increased risk of mortality.

**Conclusions:**

Among those affected early in the COVID-19 epidemic, male sex, older age, first-surge infection, and underlying medical conditions were significantly associated with mortality. Our findings are expected to provide a useful reference for future countermeasures in the early stages of pandemics involving unknown emerging infectious diseases.

## Introduction

The novel coronavirus disease 2019 (COVID-19) caused by severe acute respiratory syndrome coronavirus 2 (SARS-CoV-2) was first confirmed in December 2019 and rapidly spread worldwide, causing an explosive increase in the number of patients with COVID-19. As of August 13, 2023, the cumulative total of infected individuals worldwide was reported to be over 769 million, with a cumulative death toll of over 6.9 million ^(1)^.

**Figure 1.**
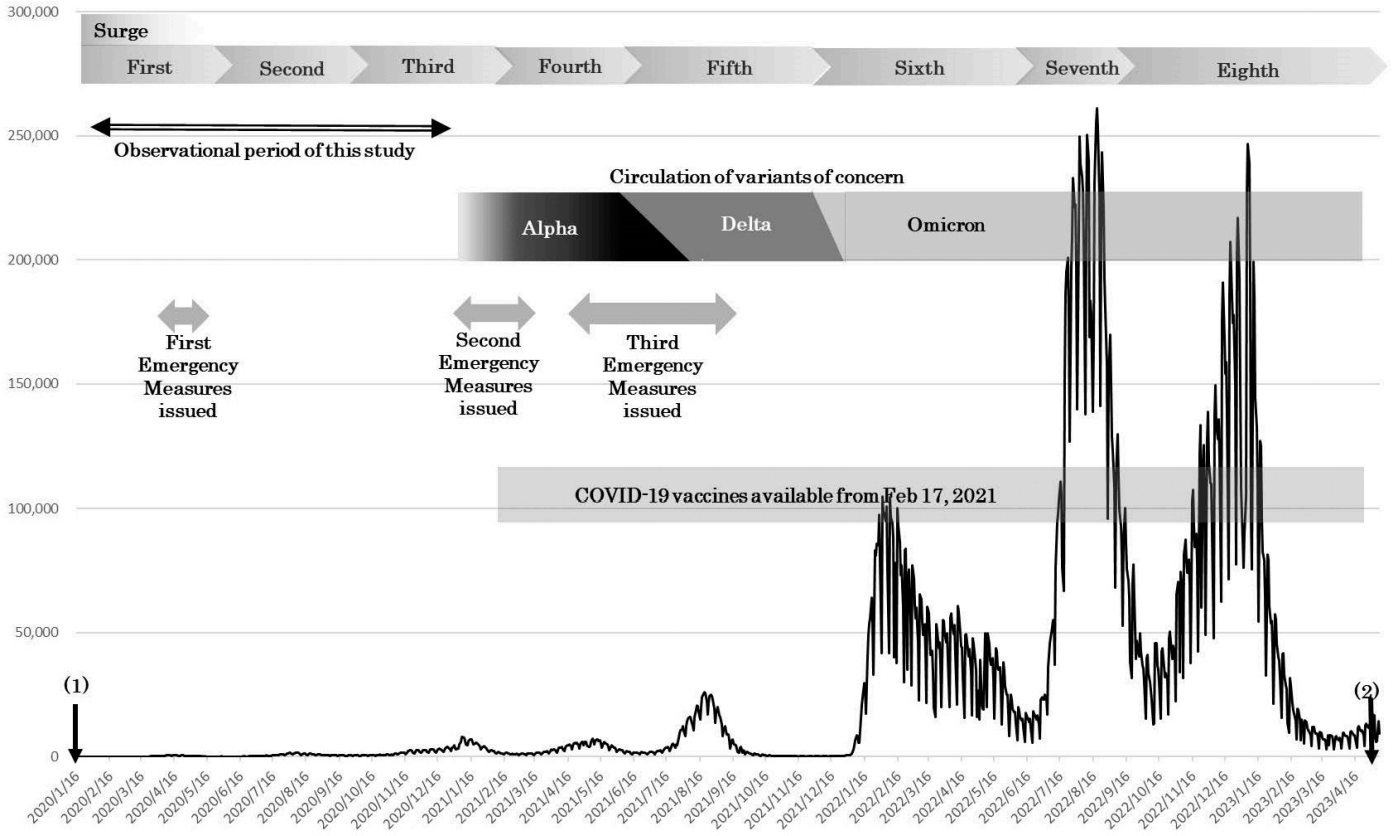
Number of reported COVID-19 cases in Japan. Drawing from the “Ministry of Health, Labour and Welfare (from January 16, 2020, to May 8, 2023) Visualizing the data: information on COVID-19 infections” (https://www.mhlw.go.jp/stf/covid-19/open-data.html). (1) The first COVID-19 patient was reported on January 16, 2020. (2) Changed to sentinel reporting for infectious disease surveillance on May 7, 2023.

**Figure 2.**
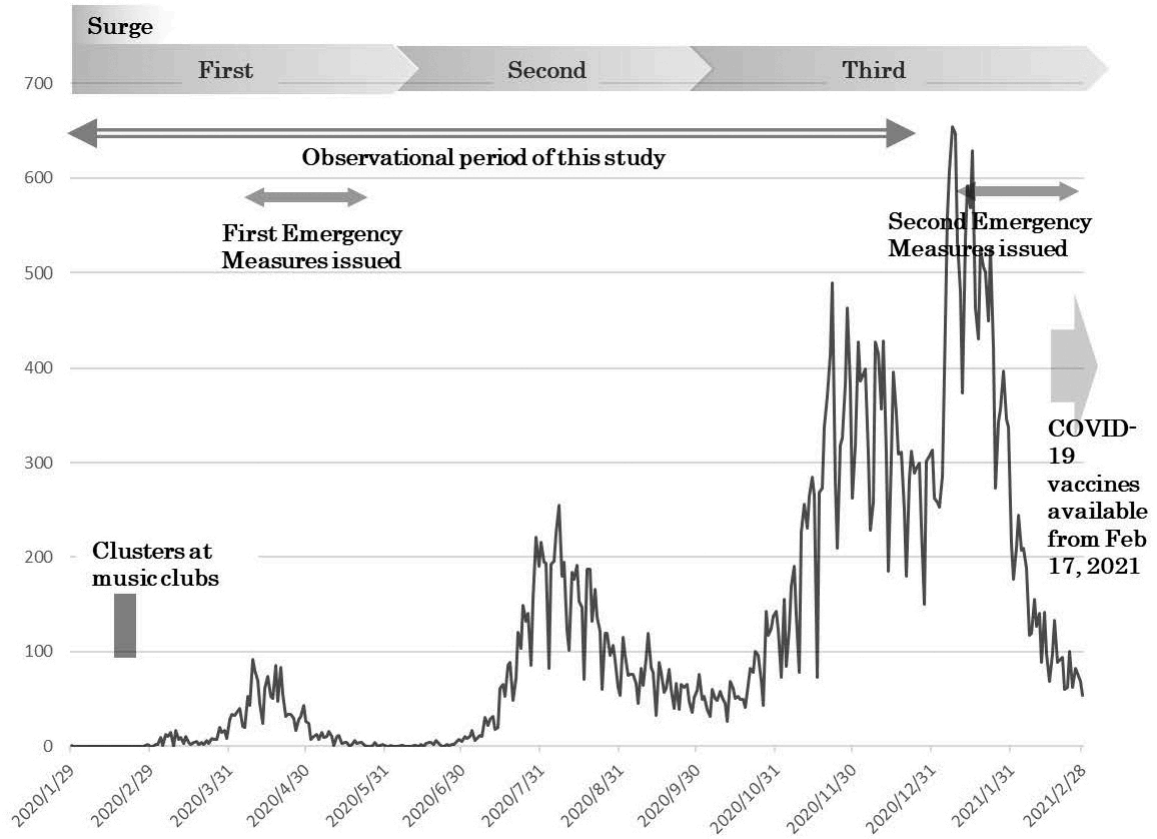
Number of COVID-19 patients reported in Osaka Prefecture, Japan (first surge: Jan 29-Jun 13, 2020; second surge: Jun 14-Oct 9, 2020; third surge: Oct 10, 2020-Feb 28, 2021), and the observational period of this study (Jan 29-Dec 24, 2020). Drawing from the “Ministry of Health, Labour and Welfare Visualizing the data: information on COVID-19 infections” (https://www.mhlw.go.jp/stf/covid-19/open-data.html).

In Japan, the first confirmed case of COVID-19 was reported on January 16, 2020, in Kanagawa Prefecture. In the late January and February 2020, many patients were reported to be among the passengers of charter flights from Wuhan, China ^(2), (3)^, and a cruise ship ^(4)^. A total of 8 surges had occurred as of May 8, 2023, when patient notifications from infectious disease surveillance were changed to sentinel reporting (Figure 1), with 33 million cumulative infections and 74,000 deaths ^(5)^. The number of cases from the first to the third surges, the period during which no COVID-19 vaccines were available, was very small. However, in addition to extreme confusion due to the frequent occurrence of clusters, strong public health measures were taken, such as declarations of state of emergency in seven prefectures. This was the situation in Osaka Prefecture, an urban prefecture with the third largest population in Japan. From February to March 2020, several clusters were identified, including attendees at music clubs ^(6)^, and a state of emergency was declared for the first time on April 7 (Figure 2).

More than 3 years have passed since the emergence of SARS-CoV-2, and several variants of concern have circulated in Japan, including Alpha, Delta, and Omicron ^(7)^. Infections with Omicron variants have been reported to result in significantly lower disease severity compared with Delta-variant infections ^(8), (9)^.

The COVID-19 pandemic far exceeded past expectations, and many government initiatives had to be started from scratch. The extraordinary experience of prefectural residents, including administrative staff in the government, should be utilized not only in responding to health and medical countermeasures for future pandemics but also in improving the medical system in the absence of emergency conditions. Therefore, analyzing the epidemiological picture in the early phase of a pandemic is important and can be informative in terms of strengthening preparedness and public health responses to infectious disease challenges that are likely to emerge in the future.

This study aimed to elucidate epidemiological aspects of the early stage of the COVID-19 pandemic in Osaka, Japan, using data accumulated by the “Osaka Prefectural Novel Coronavirus Response Status Management System” (Kintone) ^(10)^. Previous studies of the early-phase COVID-19 pandemic in Osaka Prefecture have reported that the most common symptom was high fever and that older age was associated with increased risk of mortality in patients with COVID-19 ^(11), (12), (13)^. Furthermore, a study of patients receiving extracorporeal membrane oxygenation (ECMO) reported that all patients aged ≥80 years had died ^(14)^. Thus, we aimed to elucidate factors associated with mortality, including the influence of underlying diseases, among patients with COVID-19.

## Materials and Methods

### Study design and participants

This observational study used an administrative database. The participants were patients with COVID-19 in Osaka Prefecture who were registered to Kintone from January 29 to December 24, 2020. Information on patient characteristics included sex, age, underlying diseases, and medical conditions, which have been provided to academic research institutions upon reasonable request ^(10)^. Osaka Prefecture operated Kintone, and staff in the public health centers and Department of Public Health and Medical Affairs registered patient information from the early stages of the COVID-19 pandemic. After December 25, 2020, data collection transferred to the Health Center Real-Time Information-Sharing System on COVID-19 run by the Ministry of Health, Labour and Welfare ^(15)^. Government policy on notifying the National Epidemiological Surveillance of Infectious Disease (NESID) of patients with COVID-19 has also been changed as follows: reporting was initially mandatory for all diagnosed cases and then limited to cases at a higher risk of severe disease after September 26, 2022, with weekly reporting of the number of diagnosed cases at sentinel medical institutions after May 8, 2023 ^(16)^. Thus, the patients reported during the study period included all those identified up to the middle of the third surge in Osaka Prefecture.

The study protocol was submitted to Osaka Prefecture to obtain database access for academic research purposes and was approved on February 5, 2021 (approval: Kantai no. 6099). Informed consent was not required because all information had been collected under the Infectious Diseases Law. The study protocol was also approved by the ethics committee of the Graduate School of Medicine, Osaka City University (approval no.: 2020-251). Effective April 1, 2022, Osaka City University became Osaka Metropolitan University, and the ethics committee of Osaka Metropolitan University Graduate School of Medicine has inherited all the authority of the ethics committee of the former.

### Patient information

Data included the date of health status confirmation, date of health status change, date of onset, sex, age (classified into strata of 0-9, 10-19, …, 90-99, and ≥100 years old), and underlying disease (presence/absence and details). Details of the underlying disease were coded according to the basic classifications of the International Statistical Classification of Diseases and Related Health Problems 10th Revision as follows: “Certain infectious and parasitic diseases” (A00-B99), “Neoplasms” (C00-D48), “Diseases of the blood and blood-forming organs and certain disorders involving the immune mechanism” (D50-D89), “Endocrine, nutritional, and metabolic diseases” (E00-E90), “Mental and behavioral disorders” (F00-F99), “Diseases of the nervous system” (G00-G99), “Diseases of the circulatory system” (I00-I99), “Diseases of the respiratory system” (J00-J99), “Diseases of the digestive system” (K00-K93), “Diseases of the skin and subcutaneous tissue” (L00-L99), “Diseases of the musculoskeletal system and connective tissue” (M00-M99), “Diseases of the genitourinary system” (N00-N99), “Congenital malformations, deformities and chromosomal abnormalities” (Q00-Q99), “Symptoms, signs and abnormal clinical and laboratory findings, not elsewhere classified” (R00-R99), and “Injury, poisoning and certain other consequences of external causes” (S00-T98). Data also included health status (recuperation/hospitalization/death) as of the time of registration. If a change in the health status of a patient occurred, the patient was registered again in the database with the latest status.

### Statistical analysis

Characteristics were compared using the chi-squared test, Fisher’s exact test, or Wilcoxon rank-sum test as appropriate. The follow-up period for each patient was considered as the number of days from symptom onset to health status confirmation. Because Cox proportional hazards modeling was inapplicable due to a lack of proportionality, this study employed logistic regression modeling to calculate odds ratios (ORs) and 95% confidence intervals (CIs) for each variable for mortality. The explanatory variables were sex, age, underlying disease, and time period (first, second, or third surge) based on the date of symptom confirmation. The time period was harmonized with the official statement of Osaka Prefecture, defining the first surge as January 29-June 13, 2020, the second surge as June 14-October 9, 2020, and the third surge as October 10, 2020-February 28, 2021 ^(17)^. Because of issues of data availability, analysis of the third surge was limited to data registered by December 24, 2020. For patients with missing values for the date of health status confirmation, the time period (surge) of health status confirmation was defined by date of onset or date of health status change.

Trends toward associations were evaluated by assigning an ordinal score to each category. The multivariate analysis model included sex (male/female), age (0-69/70-79/≥80 years), underlying disease (no/yes), and time period (first surge/second surge/third surge). Adjusted ORs of underlying diseases were calculated by adjusting for sex and age. Similar considerations were also made for stratified analyses by each time period.

The significance level was set to 0.05. Statistical analysis was conducted using the SAS software, version 9.4 (SAS Institute, Cary, NC, USA).

## Results

We analyzed data from a total of 14,864 patients with COVID-19 registered to the database ^(10)^. The number of patients during each period was 1,762 for the first surge, 8,744 for the second, and 4,358 for the third. According to official information from Osaka Prefecture, 36,064 patients were reported for the third surge (October 10, 2020, to February 28, 2021). Thus, data for the third surge in this study represented 12% of the total patients for that period.

Table 1 presents the characteristics of subjects. During the study period, 14,864 individuals (8,207 men, 6,657 women) with COVID-19 were registered, of whom 184 men (2.2%) and 113 women (1.7%) died (total of 297 deaths). The age-specific case fatality rate was <1% for those below 60 years old, 2.2% for those 60-69 years old, 7.8% for those 70-79 years old, 16.6% for those 80-89 years old, and about 20% for those ≥90 years old. When assessments were conducted by municipality, one of five people died in County A in Osaka Prefecture (case fatality rate, 20%). The median case fatality rate in other municipalities within Osaka Prefecture was 1.5% (interquartile range, 0.6%-2.7%). COVID-19 patients with underlying diseases exhibited a higher case fatality rate than those without. For evaluations by time period, the first surge had the highest case fatality rate (4.9% in the first surge, 1.6% in the second, and 1.7% in the third). There were 7 patients with missing health status confirmation date and 1,282 patients with missing onset date (including 3 patients missing health status confirmation date and onset date). The interval from onset to health status confirmation (days) was thus missing for 1,286 patients. The median number of days (follow-up period) from symptom onset to health status confirmation for each patient was 11 days. There were 4,404 hospitalized patients. The reported treatments included oxygen therapy (n = 792), intubation (n = 242), mechanical ventilation (n = 209), admission to an intensive care unit (n = 204), dialysis (n = 12), and ECMO (n = 37). Table 2 shows factors associated with mortality. The adjusted OR for mortality was higher in men (OR = 2.00, 95% CI = 1.54-2.60) than in women. Compared with those <70 years old, the adjusted OR was 25.4 (95% CI = 16.8-38.2) for those 70-79 years old and 78.1 (95% CI = 53.3-114) for those ≥80 years old (*P* for trend < 0.001). Patients with underlying diseases (OR = 1.74, 95% CI = 1.34-2.27) exhibited a higher OR than those without. OR decreased in the second surge (OR = 0.42, 95% CI = 0.31-0.57) and third surge (OR = 0.41, 95% CI = 0.29-0.58) compared with the first surge.

**Table 1.** Comparison of Patient Characteristics.

	All	Death (n = 297)		Alive (n = 14,567)		*P* value^a^
	(N = 14,864)	n	(%)	n	(%)	
Sex						
Male	8,207	184	(2.2)	8,023	(97.8)
Female	6,657	113	(1.7)	6,544	(98.3)	0.018
Age (years)						
0-9	329	0	(0)	329	(100)
10-19	911	0	(0)	911	(100)
20-29	4,248	0	(0)	4,248	(100)
30-39	2,281	0	(0)	2,281	(100)
40-49	2,029	3	(0.2)	2,026	(99.9)
50-59	1,857	7	(0.4)	1,850	(99.6)
60-69	1,143	25	(2.2)	1,118	(97.8)
70-79	1,066	83	(7.8)	983	(92.2)
80-89	751	125	(16.6)	626	(83.4)
90-99	239	52	(21.8)	187	(78.2)
100-	10	2	(20.0)	8	(80.0)	<0.0001
0-69	12,798	35	(0.3)	12,763	(99.7)
70-79	1,066	3	(7.8)	983	(92.2)
80-	1,000	179	(17.9)	821	(82.1)	<0.0001
Underlying diseases						
No	12,825	164	(1.3)	12,661	(98.7)
Yes	2,039	133	(6.5)	1,906	(93.5)	<0.0001
Certain infectious and parasitic diseases						
No	14,845	297	(2.0)	14,548	(98.0)
Yes	19	0	0	19	(100)	1.000
Neoplasms						
No	14,645	274	(1.9)	14,371	(98.1)
Yes	219	23	(10.5)	196	(89.5)	<0.0001
Diseases of the blood and blood-forming organs and certain disorders involving the immune mechanism						
No	14,756	286	(1.9)	14,470	(98.1)
Yes	108	11	(10.2)	97	(89.8)	<0.0001
Endocrine, nutritional, and metabolic diseases						
No	14,334	240	(1.7)	14,094	(98.3)
Yes	530	57	(10.8)	473	(89.3)	<0.0001
Mental and behavioral disorders						
No	14,842	295	(2.0)	14,547	(98.0)
Yes	22	2	(9.1)	20	(90.9)	0.071
Diseases of the nervous system						
No	14,820	294	(2.0)	14,526	(98.0)
Yes	44	3	(6.8)	41	(93.2)	0.057
Diseases of the circulatory system						
No	14,370	248	(1.7)	14,122	(98.3)
Yes	494	49	(9.9)	445	(90.1)	<0.0001
Diseases of the respiratory system						
No	14,326	265	(1.9)	14,061	(98.2)
Yes	538	32	(6.0)	506	(94.1)	<0.0001
Diseases of the digestive system						
No	14,763	294	(2.0)	14,469	(98.0)
Yes	101	3	(3.0)	98	(97.0)	0.458
Diseases of the skin and subcutaneous tissue						
No	14,861	296	(2.0)	14,565	(98.0)
Yes	3	1	(33.3)	2	(66.7)	0.059
Diseases of the musculoskeletal system and connective tissue						
No	14,843	294	(2.0)	14,549	(98.0)
Yes	21	3	(14.3)	18	(85.7)	0.008
Diseases of the genitourinary system						
No	14,716	280	(1.9)	14,436	(98.1)
Yes	148	17	(11.5)	131	(88.5)	<0.0001
Congenital malformations, deformations, and chromosomal abnormalities						
No	14,859	296	(2.0)	14,563	(98.0)
Yes	5	1	(20.0)	4	(80.0)	0.096
Symptoms, signs, and abnormal clinical and laboratory findings, not elsewhere classified						
No	14,863	297	(2.0)	14,566	(98.0)
Yes	1	0	(0.0)	1	(100.0)	1.000
Injury, poisoning, and certain other consequences of external causes						
No	14,859	295	(2.0)	14,564	(98.0)
Yes	5	2	(40.0)	3	(60.0)	0.004
Time period						
First surge (Jan 29-Jun 13, 2020)	1,762	86	(4.9)	1,676	(95.1)
Second surge (Jun 14-Oct 9, 2020)	8,744	136	(1.6)	8,608	(98.4)
Third surge (Oct 10 to Dec 24, 2020)	4,358	75	(1.7)	4,283	(98.3)	<0.0001
Time from onset to health status confirmation (days)						
Median (interquartile range)	11 (10-16)	16 (11-26)		11 (10-16)		<0.0001
Missing	1286^b^	30^c^		1256^d^	

^a^ Chi-squared test, Fisher’s exact test, or Wilcoxon’s rank-sum test ^b^ There were 7 patients with missing health status confirmation date and 1,282 patients with missing onset date (including 3 patients missing both health status confirmation date and onset date). Therefore, the interval from onset to health status confirmation (days) was missing for 1,286 patients. ^c^ There were 30 patients with missing onset date. ^d^ There were 7 patients with missing health status confirmation date and 1,252 patients with missing onset date (including 3 patients missing both health status confirmation date and onset date). Therefore, the interval from onset to health status confirmation (days) was missing for 1,256 patients. Some totals for “%” do not equal to 100% due to rounding off.

**Table 2.** Odds Ratios of Each Factor for Death.

	Crude OR (95%CI)		*P* value	Adjusted OR^a^ (95%CI)		*P* value
Sex
Male	1.33	(1.05-1.68)	0.019	2.00	(1.54-2.60)	<0.001
Female	1			1
Age (years)
0-69	1			1
70-79	30.8	(20.6-45.9)	<0.001	25.4	(16.8-38.2)	<0.001
80-	79.5	(55.0-115)	<0.001	78.1	(53.3-114)	<0.001
		Trend P < 0.001			Trend P < 0.001
Underlying diseases
No	1			1
Yes	5.39	(4.26-6.81)	<0.001	1.74	(1.34-2.27)	<0.001
Time period
First surge (Jan 29-Jun 13, 2020)	1			1
Second surge (Jun 14-Oct 9, 2020)	0.31	(0.23-0.41)	<0.001	0.42	(0.31-0.57)	<0.001
Third surge (Oct 10-Dec 24, 2020)	0.34	(0.25-0.47)	<0.001	0.41	(0.29-0.58)	<0.001

^a^ The model includes all variables in the table.

Table 3 presents the adjusted ORs of underlying diseases. The adjusted ORs of “Diseases of the blood and blood-forming organs and certain disorders involving immune mechanism” (OR = 2.98, 95% CI = 1.44-6.14), “Endocrine, nutritional and metabolic diseases” (OR = 1.95, 95% CI = 1.39-2.75), and “Diseases of the respiratory system” (OR = 1.67, 95% CI = 1.09-2.55) significantly increased compared with the absence of each category of underlying diseases.

**Table 3.** Odds Ratio of Underlying Diseases for Death.

	Crude OR (95%CI)		*P* value	Adjusted OR^a^ (95%CI)		*P* value
Neoplasms						
No	1			1
Yes	6.16	(3.93-9.64)	<0.001	1.26	(0.77-2.04)	0.359
Diseases of the blood and blood-forming organs and certain disorders involving the immune mechanism						
No	1			1
Yes	5.74	(3.05-10.8)	<0.001	2.98	(1.44-6.14)	0.003
Endocrine, nutritional, and metabolic diseases						
No	1			1
Yes	7.08	(5.23-9.58)	<0.001	1.95	(1.39-2.75)	<0.001
Mental and behavioral disorders						
No	1			1
Yes	4.93	(1.15-21.2)	0.032	1.41	(0.29-6.85)	0.671
Diseases of the nervous system						
No	1			1
Yes	3.62	(1.11-11.7)	0.033	1.47	(0.42-5.20)	0.548
Diseases of the circulatory system						
No	1			1
Yes	6.27	(4.55-8.64)	<0.001	1.30	(0.92-1.85)	0.143
Diseases of the respiratory system						
No	1			1
Yes	3.36	(2.30-4.90)	<0.001	1.67	(1.09-2.55)	0.018
Diseases of the digestive system						
No	1			1
Yes	1.51	(0.48-4.78)	0.487	0.53	(0.16-1.79)	0.310
Diseases of the skin and subcutaneous tissue						
No	1			1
Yes	24.6	(2.23-272)	0.009	5.43	(0.35-85.2)	0.228
Diseases of the musculoskeletal system and connective tissue						
No	1			1
Yes	8.25	(2.42-28.2)	0.001	4.36	(0.98-19.4)	0.054
Diseases of the genitourinary system						
No	1			1
Yes	6.69	(3.98-11.2)	<0.001	1.58	(0.88-2.84)	0.124

^a^ Adjusted for sex, age, and time period

Next, we calculated adjusted ORs for mortality by each time period (first, second, and third surges) (Table 4 and 5). ORs were significantly higher for men in the second surge (OR = 2.67, 95% CI = 1.81-3.93) and third surge (OR = 1.91, 95% CI = 1.16-3.16), but no significant difference in sex was observed in the first surge. The age-specific adjusted OR increased with age regardless of the period (*P* for trend < 0.001), with ORs for age ≥80 years showing a marked increase in the second surge (OR = 105, 95% CI = 61.2-182) and third surge (OR = 171, 95% CI = 60.3-484). Subjects with underlying diseases exhibited a significant increase in OR during the first surge (OR = 3.60, 95% CI = 2.11-6.15) and third surge (OR = 1.72, 95% CI = 1.03-2.88) but not during the second surge. Details of underlying diseases that demonstrated significant associations with mortality differed depending on the period. In the first surge, the ORs for “Diseases of blood and blood-forming organs and certain disorders involving immune mechanisms” (OR = 6.93, 95% CI = 1.48-32.4), “Endocrine, nutritional and metabolic disorders” (OR = 3.34, 95% CI = 1.93-5.77), and “Diseases of the genitourinary system” (OR = 4.82, 95% CI = 1.81-12.8) significantly increased. In the second surge, only “Endocrine, nutritional and metabolic disorders” displayed a significant increase (OR = 1.77, 95% CI = 1.03-3.06). In the third surge, the ORs of “Diseases of blood and blood-forming organs and certain disorders involving immune mechanisms” (OR = 3.86, 95% CI = 1.16-12.8) and “Diseases of the respiratory system” (OR = 2.38, 95% CI = 1.11-5.06) significantly increased.

**Table 4.** Adjusted Odds Ratios of Each Factor for Death by Time Period.

	First surge (Jan 29-Jun 13, 2020)					Second surge (Jun 14-Oct 9, 2020)					Third surge (Oct 10-Dec 24, 2020)				
	Death	/	Total participants	OR^a^ (95%CI)		Death	/	Total participants	OR^a^ (95%CI)		Death	/	Total participants	OR^a^ (95%CI)	
	(n = 86)		(n = 1,762)			(n = 136)		(n = 8,744)			(n = 75)		(n = 4,358)		
Sex															
Male	53	/	963	1.23	(0.74-2.04)	89	/	4,930	2.67	(1.81-3.93)	42	/	2,314	1.91	(1.16-3.16)
Female	33	/	799	1		47	/	3,814	1		33	/	2,044	1	
Age (years)															
0-69	14	/	1,446	1		17	/	7,663	1		4	/	3,689	1	
70-79	29	/	168	13.3	(6.67-26.6)	29	/	539	24.8	(13.4-45.9)	25	/	359	61.8	(21.2-180)
80-	43	/	148	26.3	(13.5-51.3)	90	/	542	105	(61.2-182)	46	/	310	171	(60.3-484)
				Trend P<0.001					Trend P < 0.001					Trend P < 0.001	
Underlying diseases															
No	25	/	1,369	1		91	/	7,657	1		48	/	3,799	1	
Yes	61	/	393	3.60	(2.11-6.15)	45	/	1,087	1.24	(0.84-1.85)	27	/	559	1.72	(1.03-2.88)

^a^ The model includes all variables in the table.

**Table 5.** Odds Ratios of Underlying Disease for Death by Time Period.

	First surge (Jan 29-Jun 13, 2020)					Second surge (Jun 14-Oct 9, 2020)					Third surge (Oct 10-Dec 24, 2020)				
	Death	/	Total participants	OR^a^ (95%CI)		Death	/	Total participants	OR^a^ (95%CI)		Death	/	Total participants	OR^a^ (95%CI)	
	(n = 86)		(n = 1,762)			(n = 136)		(n = 8,744)			(n = 75)		(n = 4,358)		
Neoplasms															
No	78	/	1,718	1		126	/	8,647	1		70	/	4,280	1
Yes	8	/	44	1.48	(0.61-3.58)	10	/	97	1.27	(0.61-2.62)	5	/	78	1.04	(0.39-2.76)
Diseases of the blood and blood-forming organs and certain disorders involving the immune mechanism															
No	82	/	1,747	1		133	/	8,691	1		71	/	4,318	1
Yes	4	/	15	6.93	(1.48-32.4)	3	/	53	1.58	(0.45-5.60)	4	/	40	3.86	(1.16-12.8)
Endocrine, nutritional, and metabolic diseases															
No	55	/	1,619	1		116	/	8,516	1		69	/	4,199	1
Yes	31	/	143	3.34	(1.93-5.77)	20	/	228	1.77	(1.03-3.06)	6	/	159	0.86	(0.36-2.10)
Mental and behavioral disorders																	
No	86	/	1,756	NA		134	/	8,730	1		75	/	4,356	NA	
Yes	0	/	6			2	/	14	1.78	(0.34-9.22)	0	/	2		
Diseases of the nervous system															
No	86	/	1,753	NA		133	/	8,716	1		75	/	4,351	NA	
Yes	0	/	9			3	/	28	1.87	(0.49-7.12)	0	/	7		
Diseases of the circulatory system																	
No	64	/	1,646	1		122	/	8,525	1		62	/	4,199	1
Yes	22	/	116	1.58	(0.88-2.84)	14	/	219	0.96	(0.53-1.76)	13	/	159	1.60	(0.83-3.09)
Diseases of the respiratory system															
No	77	/	1,661	1		123	/	8,511	1		65	/	4,154	1
Yes	9	/	101	1.24	(0.55-2.79)	13	/	233	1.65	(0.87-3.16)	10	/	204	2.38	(1.11-5.06)
Diseases of the digestive system															
No	85	/	1,748	1		134	/	8,699	1		75	/	4,316	NA	
Yes	1	/	14	0.90	(0.10-8.50)	2	/	45	0.70	(0.16-3.16)	0	/	42		
Diseases of the skin and subcutaneous tissue															
No	85	/	1,760	NA		136	/	8,743	NA		75	/	4,358	NA	
Yes	1	/	2			0	/	1			0	/	0		
Diseases of the musculoskeletal system and connective tissue															
No	84	/	1,748	1		136	/	8,739	NA		74	/	4,356	NA	
Yes	2	/	14	2.62	(0.46-14.8)	0	/	5			1		2		
Diseases of the genitourinary system															
No	76	/	1,727	1		133	/	8,675	1		71	/	4,314	1
Yes	10	/	35	4.82	(1.81-12.8)	3	/	69	0.56	(0.16-1.91)	4	/	44	1.42	(0.46-4.36)

NA, not applicable. ^a^ Adjusted by sex and age.

## Discussion

This study found that among those affected in the early stages of the COVID-19 pandemic in Osaka, male sex, older age, early infection, and the presence of underlying diseases were significantly associated with mortality. The underlying disease classes showing significant associations with mortality differed depending on the period. These findings would be valuable in terms of epidemiological evaluations for the period before the distribution of COVID-19 vaccines.

As of May 7, 2023, Osaka Prefecture had the second highest number of COVID-19 patients after Tokyo. The cumulative number of COVID-19 patients in Osaka Prefecture reached 2.8 million (approximately 10% of the total number of COVID-19 patients in Japan), and about 8500 patients died (approximately 11.5% of the total number of patient deaths due to COVID-19 in Japan) ^(5)^. In this study, we were able to analyze case fatality rates among all first- and second-surge registrants, and among limited registrants from the third surge (until December 24, 2020). The case fatality rate for the third surge among the subjects in this study was 1.7%, but according to the official information from Osaka Prefecture, the case fatality rate for the entirety of the third surge (October 10, 2020, to February 28, 2021) was 2.6% ^(17)^. Therefore, it is noteworthy that the case fatality rate in the third surge was underestimated in the present study.

Regarding adjusted ORs for mortality, no sex differences were observed in the first surge, but adjusted ORs were significantly higher for men in the second and third surges. Many studies have investigated sex differences in COVID-19 infection, including a meta-analysis from December 1, 2019, to May 31, 2020 ^(18)^, a retrospective cohort study in the United States ^(19)^, and an analysis of electronic medical records in the United States ^(20)^, all finding that male sex was associated with higher mortality from COVID-19. Epidemiological studies in Japan ^(21), (22)^ have also reported that male sex was associated with invasive ventilation or death from COVID-19. Although the mechanisms remain unclear, men may be more susceptible to COVID-19.

The case fatality rate was less than 1% in those <60 years old but increased with age. In each of the first, second, and third surges, the ORs after adjusting for other factors were significantly higher in those ≥80 years old than in those <70 years old. Older age has been associated with more serious outcomes in national and international reports ^(11), (12), (13), (14), (18), (19), (20), (21), (22), (23)^.

In this study, the ORs increased among patients with underlying disease compared with those without. When analyzing details in diseases, the ORs of “Diseases of the blood and blood-forming organs and certain disorders involving immune mechanism” in the first and third surges, “Endocrine, nutritional and metabolic diseases” in the first and second surges, and “Diseases of the respiratory system” in the third surge significantly increased. The OR of “Diseases of the genitourinary system” also significantly increased in the first surge. A large-scale cohort study using data registered to the COVID-19 Registry Japan (COVIREGI-JP) targeted hospitalized patients at 802 hospitals in Japan. They examined the severity and risk factors for disease progression during hospitalization and demonstrated that cerebrovascular disease, liver disease, renal disease or dialysis, solid tumors, and hyperlipidemia all influenced mortality ^(23)^. In Japan, doctors diagnosing COVID-19 patients immediately notify the local public health center according to the Infectious Diseases Control Law. Then, the public health center then submits the collected data to the NESID. In a study using NESID data from January to March 2020, diabetes, dyslipidemia, hyperuricemia, and lung disease were associated with severe outcomes ^(21)^. A literature review and meta-analysis of COVID-19 patient data up to March 20, 2020, revealed that hypertension, diabetes, cardiovascular disease, and respiratory disease can significantly impact the prognosis of COVID-19 ^(24)^. An analysis of US electronic medical records between January 20 and May 26, 2020, revealed that myocardial infarction, congestive heart failure, dementia, chronic lung disease, mild liver disease, moderate/severe liver disease, renal disease, and metastatic solid tumors as pathologies were significantly associated with increased risk of mortality among adults with COVID-19 ^(20)^. Findings from our study, which broadly resembled the results of those studies, indicate the importance of paying attention to patients with “Diseases of blood and blood-forming organs and certain disorders involving immune mechanisms,” “Endocrine, nutritional and metabolic diseases,” “Diseases of the respiratory system,” and “Diseases of the genitourinary system” in the early stages of the pandemic, regardless of sex or age.

This study reported several limitations that need to be acknowledged. First, we considered patients for whom death information was identified on the database as an outcome occurrence, but these deaths were not necessarily attributable to COVID-19 itself. Second, because information on underlying diseases was entered under ICD-10 basic classifications, we were unable to examine the associations between disease subdivisions and death in patients with COVID-19. Furthermore, the variables that could be used were limited due to the open nature of the data. Third, although information on underlying diseases was based on notifications from medical institutions, we cannot rule out the possibility that data accuracy may have been compromised due to the chaos at the frontline during the COVID-19 pandemic. Finally, the inclusion of third-surge deaths in this study was incomplete due to the transition between management systems in Osaka Prefecture during the study period.

In conclusion, factors associated with mortality among the affected individuals early in the COVID-19 pandemic were male sex, older age, underlying conditions, and first-surge infection. Among the underlying diseases, “Diseases of blood and blood-forming organs and certain disorders involving immune mechanism,” “Endocrine, nutritional and metabolic disorders,” “Diseases of the respiratory system,” and “Diseases of the genitourinary system” were associated with increased risk of mortality. We hope that our results will provide useful reference data for future countermeasures in the early stages of pandemics involving unknown emerging infectious diseases.

## Article Information

### Conflicts of Interest

None

### Acknowledgement

We would like to thank all staff at the Osaka Prefectural Government and Public Health Centers in Osaka Prefecture for their efforts in collecting the data used in this study. We would also like to thank all medical and healthcare personnel who managed patients with COVID-19 in Osaka Prefecture.

### Author Contributions

Conceptualization: K.K., S.O., T.K., and W.F.; methodology: K.K., S.O., T.K., and W.F.; software: K.K., A.S., S.O., E.M., and W.F.; validation: all authors; formal analysis: K.K., A.S., and W.F.; investigation: all authors; resources: K.K., A.S., S.O., E.M., T.K., and W.F.; data curation: K.K., A.S., and W.F.; writing―original draft preparation: K.K.; writing―review and editing: K.K., S.O., T.K., and W.F.; visualization: K.K., S.O., E.M., T.K., and W.F.; supervision: W.F.; project administration: K.K., and W.F.; funding acquisition: none. All authors have read and agreed to the published version of the manuscript.

### Approval by Institutional Review Board (IRB)

The study protocol was also approved by the ethics committee of the Graduate School of Medicine, Osaka City University (approval no. 2020-251). Effective April 1, 2022, Osaka City University became Osaka Metropolitan University, and the ethics committee of Osaka Metropolitan University Graduate School of Medicine has inherited all authority of the ethics committee of Osaka City University Graduate School of Medicine.

### Informed Consent

The need to obtain patient consent was waived because all data were collected under the Infectious Diseases Law in Japan.
